# Supplementary figures and images for: Circulating small extracellular vesicle RNA profiling for the detection of T1a stage colorectal cancer and precancerous advanced adenoma
Source: eLife. 2024 Aug 9;12:RP88675. doi: 10.7554/eLife.88675 (PMC11315448; doi:10.7554/eLife.88675)

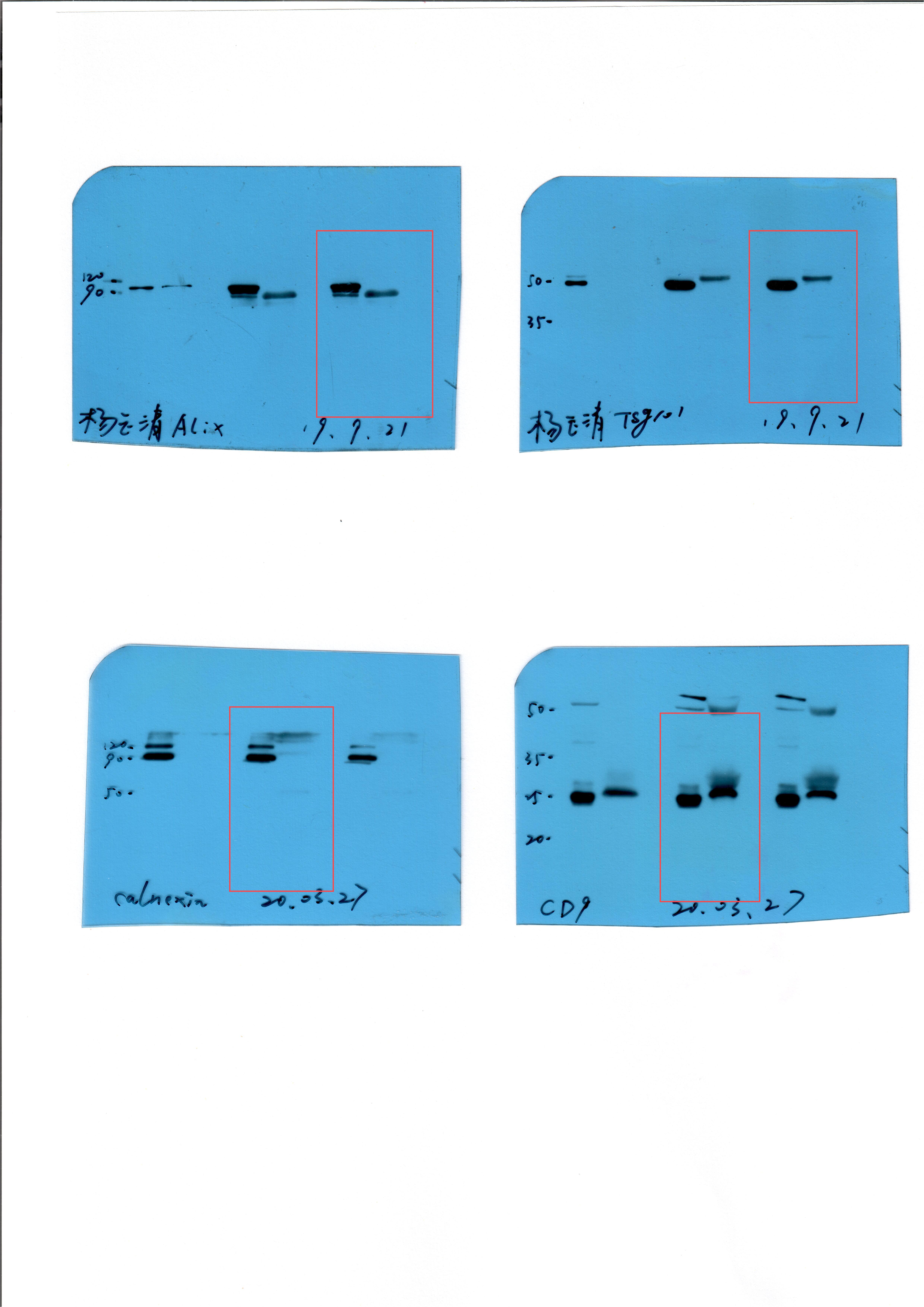

Supplement: Figure 2—source data 1. [file elife-88675-fig2-data1.zip › Figure 2-source data1.jpg]

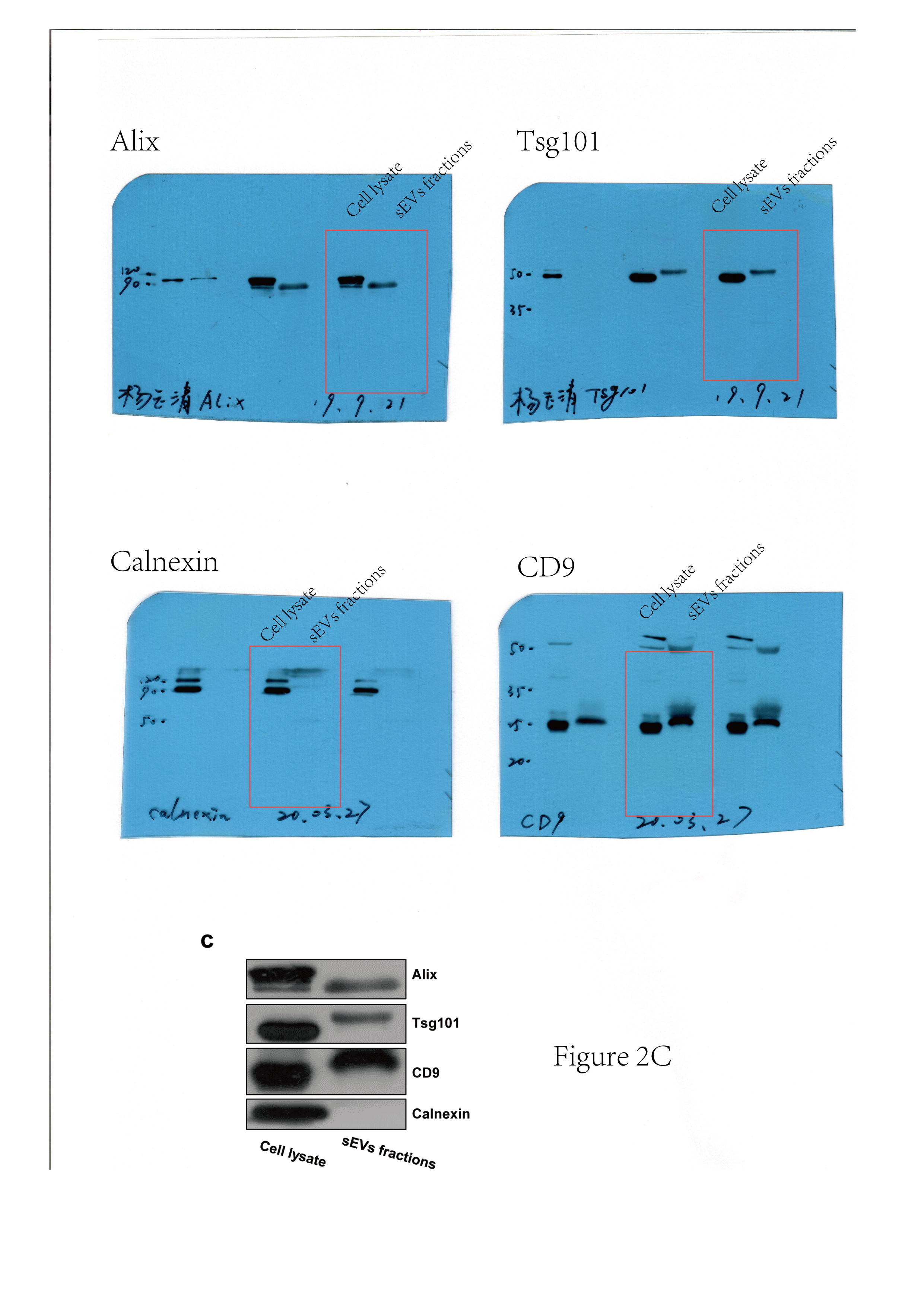

Supplement: Figure 2—source data 2. [file elife-88675-fig2-data2.zip › Figure 2-source data2.tif]
